# Supplementary material for: “My friends said they should no longer be with a b*tch like me…”: A qualitative study to explore the consequences of adolescent childbearing among teen mothers in Gatsibo district, Rwanda
Source: PLOS Glob Public Health. 2024 Sep 11;4(9):e0003716. doi: 10.1371/journal.pgph.0003716 (PMC11389937; doi:10.1371/journal.pgph.0003716)
Supplement: Attachment — Submitted filename: Manuscript_Innocent_rev.docx [file pgph.0003716.s001.docx]

**“…My friends said they should no longer be with a b*tch like me…”: A qualitative study to explore the consequences of adolescent childbearing among teen mothers in Gatsibo district, Rwanda**

Innocent Twagirayezu ^1,2*^, Joselyne Rugema ^1^, Aimable Nkurunziza ^1,2^, Alice Nyirazigama ^1,2^, Vedaste Bagweneza ^1^, Belancille Nikuze^1^, Jean Pierre Ndayisenga^1,2^

^1^ School of Nursing and Midwifery, University of Rwanda College of Medicine and Health Sciences, Kigali, Rwanda

^2^ Arthur Labatt Family School of Nursing, Western University, London, Ontario, Canada

*****Corresponding author: Innocent Twagirayezu, email: [twagiracent@gmail.com](mailto:twagiracent@gmail.com)

**ABSTRACT**

**Background**

Globally, the rate of teenage pregnancy continues to be high, with significant implications for maternal health. The prevalence of teenage pregnancy is worrisome in Rwanda and little is known about the consequences faced by teen mothers aged between 15 and 19 years. Therefore, the present study aims to explore the consequences of adolescent childbearing among teen mothers in Gatsibo district, Rwanda.

**Methods**

A descriptive qualitative study design was carried out. We purposively recruited 20 teen mothers aged between 15-19 years from four health centers in Gatsibo district for interview. The conversations were audio recorded in Kinyarwanda, verbatim translated into English, and then inductively and thematically analyzed.

**Results**

Three themes were identified: 1) self-reported health outcomes; 2) socio-economic consequences; and 3) perceived structural barriers to accessing healthcare services. Teen mothers in this study reported experiencing physical and psychological problems during pregnancy and after delivery. The socio-economic consequences included feeling guilty, lack of social support, stigma and discrimination, domestic violence, school interruptions and drop-out and financial constraints. The structural barriers to accessing healthcare included health facility mandatory requirements, being stigmatized by adult mothers in the healthcare environment, and healthcare providers’ negative attitudes towards teen mothers.

**Conclusion**

The results from this study indicated that adolescent childbearing is associated with several negative consequences. Therefore, there is a need to develop interventions addressing these consequences and revise policies and laws to ensure they support the needs of teen mothers.

**Key words:** Adolescent childbearing, teen mothers, consequences

**Background**

Adolescent pregnancy is a worldwide public health issue that affects both industrialized and developing nations (Vieira Martins et al., 2023). While some adolescent’s births and pregnancies are planned and intended, the majority are not (UNFPA, 2022). Every year, an average of 20 million adolescent girls give birth worldwide, and many of them conceive unexpectedly (Darroch et al., 2016; Worku et al., 2021). The world's highest rate of adolescent childbearing is seen in Sub-Saharan African nations with 21.5% occurring in eastern Africa where Rwanda belongs (Nkurunziza et al., 2020; Uwizeye et al., 2020; Worku et al., 2021).

Early pregnancy poses substantial risks to both the mother's and the unborn child's health (WHO, 2019). Compared to older women, adolescent females in the 15–19 age range had a twofold higher risk of dying from reasons connected to pregnancy and delivery, and their children had a 50% greater chance of dying before the age of one. Additionally, adolescent mothers are more constrained in their ability to pursue educational opportunities than young women who delay childbearing (Gurung et al., 2020; Ngoda et al., 2021; Nkurunziza et al., 2020; Uwizeye et al., 2020).

Children born to very young mothers are vulnerable to illnesses that affect children under the age of five, resulting in increased low vaccination coverage, malnutrition and infant mortality (Ajala, 2014; Gurung et al., 2020). This is mainly because teen mothers are vulnerable to poverty, lack knowledge regarding child care, and lack of support from the father of the child, their parents, and the communities in which they live (Uwizeye et al., 2020). Furthermore, it is widely documented that teenage pregnancies have greater incidences of eclampsia, puerperal endometritis, low birth weights, and premature births than pregnancies in women aged 20 to 24 (Ajayi et al., 2021; Uwizeye et al., 2020). These complications rank second among the leading causes of death among under-18 pregnancies worldwide (Kirbas et al., 2016). Stigma, family rejection, violence and school dropout, are some of the social consequences associated with adolescent pregnancy (Lebni et al., 2021; Svanemyr, 2020) and all these consequences lead to fewer employment opportunities among adolescents mothers (Gurung et al., 2020; Worku et al., 2021).

The recent Rwanda Demographic Health Survey (RDHS), 2019–2020, adolescent girls aged between 15-19 who have begun childbearing were 6.4% and this rate is higher than the overall national rate. In the same survey, Gatsibo district ranked among the top three with the overall percentage of 8.7% of teenage pregnancy rate (National Institute of Statistics of Rwanda, 2020). Despite these alarming statistics, little is known about the consequences associated with teenage pregnancies among these female adolescents to understand the full scope of the issue better. Further research is needed to inform policy decisions and interventions to support these young women. Therefore, the present study will explore the consequences of adolescent childbearing among adolescent females aged 15–19 years living in Gatsibo district.

**Methods.**

**Study design, setting, and population**

This study employed a qualitative descriptive methodology to gather rich descriptions about the consequences associated with teenage pregnancies (Creswell, 2014). The study participants included 20 childbearing adolescents from four different health centers in Gatsibo district, in the eastern province of Rwanda. We interviewed five participants who lived in the neighborhood of each health centre.

**Sampling and recruitment**

A purposive sampling strategy was used to select adolescents who gave birth when they were below 18 years old (Creswell, 2014). After getting ethical approval from University of Rwanda, we sought permission from Gatsibo Distinct to access the study settings. The permission letter was presented to the heads of the selected health centers, who connected us with community health workers (CHWs). The latter identify and register pregnant women in the communities, therefore were well positioned to know pregnant adolescents in their respective communities. We explained the study purpose to them. We left a recruitment poster with the CHWs and at the health center with our contact information so that anyone who wanted to participate could contact us and set up an appointment. We included only adolescent females aged 15-19 who got pregnant while residing in the selected communities.

**Data collection instrument**

We used a semi-structured interview guide developed from a thorough examination of the literature in the field of adolescent pregnancy (Bahamondes, 2018; Chung et al., 2018; Guttmacher Institute, 2015; Karaçam et al., 2021) and in consultation with two experienced researchers in the field. The study participants' socio-demographic characteristics, such as age, education, marital status, religion, residency, household head, parity, antenatal visit, gestational age, and occupation, were collected at the beginning of the study. The developed guide had invitational questions and probing questions that encouraged participants to express all consequences they meet individually related to childbearing. The interview was in Kinyarwanda because it was the only language our participants were able to express themselves in.

The interview guide started out with general questions and included questions such as: (1) In what circumstances did you get pregnant? (2) Can you tell me how you felt after getting pregnant, during pregnancy, and after giving birth? (3) What social problems did you experience during and after your pregnancy? (4) Did you experience any obstetrical outcomes related to getting pregnant as a teen? (5) Are there any other problems that we have not discussed?

**Data collection**

Participants who were invited through community health workers met one by one with two female co-investigators in a clean and well-ventilated room for an audio-recorded interview. The rooms were selected with the help of the administration of the health centers. Prior to the discussion, the two investigators introduced themselves to the participants. They explained the purpose of the study and provided information about the risks and benefits of participating. The participants were given time to ask questions and were provided contact information for the researchers in case of any questions or concerns. The participants were also asked to sign an assent form for those under 18 and a consent form for their legal guardians. The study participants aged 18 and over signed informed consent. The two investigators explained their role in the process of data collection, and set ground rules that encouraged respect for one another and informed conversation. One co-investigator was conducting the interview while another took field notes. Because questions were subjecting some participants to recall painful experiences related to childbearing when they were in the adolescent phase, for some, it was necessary at some point to stop the interview. Participants were counselled by both investigators, who had mental health skills. Because the topic was sensitive, there was a pre-established protocol to manage emotional responses, including stopping the interview, counselling the study participants, asking them if they wanted to continue the interview or stop, referral to mental health care professionals for further management and follow-up. Only five participants had emotional responses, such as crying and a tight throat, and wished to continue the interview. Two follow-up calls were made after one week and one month, and the participants reported they were doing well. We reached data saturation after interviewing 20 participants because there was no new information. The shortest interview was 13 minutes and 22 seconds, while the longest one was 27 minutes and 12 seconds. Data collection started August 30, 2022 and ended August 31 2022.

**Data analysis**

Data were translated into English and transcribed verbatim by IT, JR, AN, VB & BN who are fluent in Kinyarwanda. Data were organized and coded using Dedoose software. Thematic analysis was used to generate and report themes through six phases including data familiarization, generating initial codes, searching themes, reviewing themes, defining themes, and reporting themes (Braun & Clarke, 2006). Four research team members, including two data collectors, were involved in data analysis to ensure that the data was accurately interpreted and conclusions were based on the data collected. The team members reviewed the data and identified possible trends on the developed codebook through regular meetings. All team members agreed on the final themes.

**Results**

Twenty pregnant adolescents under 18, , participated in the study. Regarding socio-economic charateristcis 85% of the participants had received primary education, lived in rural areas, and were engaged in farming as their occupation. Almost all of them had experienced at least one pregnancy and had attempted to visit a clinic more than twice before giving birth (Table 1)

**Table 1. Demographic characteristics of the participants**

| **Age in years** | **Education** | **Marital status** | **Religion** | **Residency** | **Household head** | **Parity** | **Antenatal visit** | **Gestation weeks at delivery** | **Occupation** |
| --- | --- | --- | --- | --- | --- | --- | --- | --- | --- |
| 17 | Primary education | Single | Others | Urban | Male | 1 | More than 2 | 36 | Farmer |
| 17 | Secondary | Single | Protestant | Rural | Female | 1 | More than 2 | 39 | Unemployed |
| 18 | Primary education | Single | Protestant | Urban | Female | 1 | More than 2 | 36 | Unemployed |
| 18 | No education | Single | Catholic | Rural | Female | 1 | None | 37 | Student |
| 18 | Primary education | Single | Others | Rural | Female | 1 | More than 2 | 36 | Farmer |
| 17 | Primary education | Single | Protestant | Rural | Male | 1 | Between 1 and 2 | 36 | Unemployed |
| 17 | Primary education | Single | Protestant | Rural | Male | 0 | Between 1 and 2 | 37 | Farmer |
| 15 | No education | Single | Catholic | Rural | Female | 1 | Between 1 and 2 | 39 | Farmer |
| 16 | Primary education | Single | Protestant | Rural | Female | 1 | Between 1 and 2 | 39 | Student |
| 17 | Primary education | Single | Others | Rural | Male | 1 | Between 1 and 2 | 36 | Farmer |
| 18 | Primary education | Single | Catholic | Rural | Female | 1 | More than 2 | 40 | Farmer |
| 17 | Primary education | Single | Protestant | Rural | Female | 1 | More than 2 | 40 | Farmer |
| 17 | No education | Single | Catholic | Rural | Female | 1 | More than 2 | 39 | Farmer |
| 18 | Primary education | Single | Catholic | Rural | Female | 1 | More than 2 | 40 | Farmer |
| 15 | Primary education | Single | Protestant | Rural | Male | 1 | More than 2 | 39 | Farmer |
| 18 | Secondary education | Single | Protestant | Rural | Female | 1 | More than 2 | 39 | Farmer |
| 17 | Secondary  education | Single | Catholic | Rural | Female | 1 | More than 2 | 39 | Farmer |
| 17 | Secondary education | Single | Protestant | Rural | Male | 1 | Between 1 and 2 | 30 | Farmer |
| 15 | Primary education | Single | Protestant | Rural | Female | 1 | More than 2 | 40 | Unemployed |
| 17 | Secondary education | Single | Protestant | Urban | Female | 1 | More than 2 | 40 | Farmer |

**Themes**

Three main themes were identified during thematic analysis: a) self-reported health outcomes; b) socio-economic consequences; and c) perceived structural barriers to access healthcare services (table 2).

**Table 2: Themes and sub-themes**

| **Themes** | **Sub-themes** |
| --- | --- |
| **Self-reported health outcomes** | Physical |
|  | Psychological |
| **Socio-economic consequences** | Loss of future |
|  | Lack of social support |
|  | Stigma and discrimination |
|  | Domestic violence |
|  | School interruptions and drop-out |
|  | Financial constraints |
| **Perceived structural barriers to access healthcare services** | Health facility mandatory requirements |
|  | Adult mothers’ stigma in a healthcare environment |
|  | Healthcare providers’ negative attitudes towards teen mothers |

**Theme one: Self-reported health outcomes**

Some teen mothers in this study reported experiencing physical and psychological problems during pregnancy and after delivery. Physical problems included prolonged labor, pain; some said their skin turned yellow to mean anemia. Psychological problems included fear of disclosure, intense stress, anxiety, and depression, feeling guilty, and poor coping mechanisms (sleeplessness, suicidal thoughts, committing suicide, and abortion).

A few of them had premature deliveries, others underwent caesarian section, there are those that had anemia during pregnancy, and a very small number reported high blood pressure.

For instance, one teen mother noted, “*Throughout my pregnancy, I was weak, my skin was always yellow and that left me always feeling worn out”* Site 4, P2.

Another participant added “*I was referred immediately from the health center to the district hospital because my blood pressure was high and told that it would be severe.”* Site 4, P5.

A few participants experienced mental problems including accepting that they were pregnant, fear of disclosure, intense stress, anxiety and depression related to the delivery process and relationships with their family members when they knew they were pregnant.

One participant explained, "After learning that I was pregnant, I felt overwhelmed with despair but eventually accepted the reality of the situation" Site 4, P5.

Other participants expressed that, even though they accepted to give birth, they experienced fear and intense stress related to pregnancy and delivery process as noted in one quote, "The fear I experienced was specifically related to the delivery process and unprepared parenthood" Site 1, P1.

Another participant added, "I was filled with fear and apprehension regarding my father's reaction upon learning about my pregnancy" Site 3, P2.

Most of the study participants narrated that after learning that they were pregnant, they felt guilty and ashamed. For instance, a study participant explained, *"I deeply regretted getting pregnant at my age, as it was an unplanned occurrence. It was a shame on my family."* Site 1, P3

A few study participants reported that these psychological problems led to poor coping mechanisms. The study participants reported that they experienced difficulties coping with the situation, and some of them could spend sleepless nights, as stated by one study participant:

*“I felt depressed when I called the father of this child and found that the phone was off. After being discharged from hospital, I found it [phone] inaccessible. I could not sleep for a long time”* Site 1, P1.

A few other participants had suicidal thoughts and others tried to commit suicide as reflected in the following quote:

I attempted suicide using a lace, but during the act, I felt physical pain in my body, including headaches and earaches. The lace broke and fell off. On another occasion, I tried to end my life by going to Lake Muhazi, which was far from my home. However, while on my way, it started raining heavily, and I thought about my child being left alone at home. I quickly turned back and rushed home, where I found my child crying. Seeing my child in that state, I was overwhelmed with emotion, sought his or her forgiveness, and made a commitment to never engage in such actions again. Site 4, P5

A few other participants reported that due to the lack of social support and being ostracized by their families, they did not want to raise their babies. They felt abortion was a solution. For instance, one participant explained:

Given the circumstances, I was facing, my family decided to disown me, leaving me homeless. Throughout that difficult period, the idea of having an abortion crossed my mind. However, my conscience strongly opposed the notion. I struggled with these conflicting thoughts until I ultimately made the decision to give birth. Site 1, P2

The study participants reported that teenage pregnancy was associated with a variety of health problems, such as mental health problems, and poor physical health. This is likely due to the fact that teenage mothers often lack the support and resources needed to take care of their health.

**Theme two: Socio-economic consequences**

The study participants in this study reported various socio-economic consequences of teenage pregnancy at individual and society levels. These consequences included loss of future, lack of social support, stigma and discrimination, family conflict, school interruptions and drop-out and financial constraints.

Most of the study participants narrated that after learning that they were pregnant, they felt disappointed in their families, and others felt their dreams shattered. For instance, a study participant explained, *"It was a very difficult time for me as I was worried about the future of my child... I couldn't help but think about my own life. …. It was also hard for me to see my colleagues going back to school while I had to put my education on hold due to the pregnancy."* Site 3, P2

After getting pregnant or after delivery, teen mothers in this study reported lacking support from their partners, and families. Most study participants reported being abandoned by their partners. For instance, one study participant explained, *"After engaging in sexual intercourse with my boyfriend and revealing my pregnancy, he informed me that I should have an abortion as he wasn't ready for marriage. Thus, he did not support me."* Site 4, P3 Another participant confirmed, "*I was left alone by the man who had impregnated me, without any support or even providing the necessary materials as the delivery approached. This caused me a lot of stress and frustration, especially when I found out that he had gotten married to another woman."* Site 2, P2

Teen mothers expressed encountered family problems, their pregnancy is essence family conflicts such quarrels, misunderstandings between parents, family separation, family conflicts were found to be the first source of psychological problems of teen mothers after getting pregnant. Some families never accept their child even grandchild, most the teen fathers are the first to traumatize their daughters but few mothers also have been found, but on other side family support was the pillar of their wellbeing and their psychological stability of teen mothers, not only family conflicts of teen mothers but extent to the family of the biological father of the baby.

He [my father] was very harsh to me, to the extent that I lost my appetite due to his words. Even before, when he used to drink, he would treat me harshly by claiming that he didn't give birth to a child like me and would speak unfriendly words to my mother, accusing her of my problems. It became difficult for me to eat in such a situation. Site 2, P1

*Because my family didn’t accept my situation of being pregnant, it was a big challenge because no one cared about me. Even when I need something, they said to go and seek assistance from the responsible for the pregnancy, while I was not aware of where he was.* Site 1, P5

Some study participants revealed that they were stigmatized and discriminated by their peers and the entire community as a result of teenage pregnancy. Among 20 teen mothers interviewed 15 of them faced challenges to separate with their peers, childhood friends, schoolmates, some consider them becoming prostitutes, misbehaved girls, they talk on their back saying none can give good advices as they got pregnant early.

For example, one participant noted, *“My friends said they should no longer be with a bitch like me. They claimed that I could influence others negatively.”* Site 1, P3

Another participant added, *“They broke up our relationship and shit me claiming that I am no longer a girl but woman”* Site 4, P2

All 20 interviewed teen mothers dropped out from schools, many of them got pregnant while they were still studying, fewest got pregnant while already are no longer studying owed to different reasons, only one teen mother wished to get back to school after 2 years of breastfeeding. For instance, one study participant highlighted, *“After getting pregnant, I became less interested in school activities. Finally, I dropped out.”* Site 1, P4

Lastly, teen mothers in this study reported some financial constraints as a result of getting pregnant. These long-term economic consequences were due to lack of employment opportunities, stable jobs, and childcare responsibilities, as reflected in the following quote:

*You could have money and wish to buy something, but inevitably, you think about your child, my child lacks shoes, if I could buy them, how would I go to vaccination appointments…, I am going there without taking porridge or without shawl of my baby? without a baby umbrella then you think deeply about your life situation and realize that the reality of life changes.* Site 3, P5

This was also confirmed by another participant who explained, *“Having a child made it really difficult for me to complete my education and enroll in college. Finding a job without a diploma has proven difficult for me”* Site 4, P2*.* Other reported that the hiring people could not hire them due to their pregnancy status as one participant noted, *“…No one can give you a job when you are pregnant because they fear that you might fall and get injured. As a result, you lose the money needed to buy things like baby clothes”* Site 3, P3.

Understandably, adolescents in this study faced several socio-economic consequences resulting from the loss of opportunities such as education and jobs. These consequences included poverty, family instability, and difficulty in accessing resources. This lack of opportunities profoundly impacted adolescents' ability to plan for the future, realize their dreams, develop self-efficacy, and gain access to the resources needed to succeed.

**Theme three: Perceived structural barriers to access healthcare services**

All study participants interviewed pointed out some barriers faced by teen mothers. These barriers included health facility mandatory requirements, being stigmatized by adult mothers in the healthcare environment, and healthcare providers’ negative attitudes towards teen mothers.

They are challenged by policies and procedures at the health centre for the first antenatal care visit, where they are required to be accompanied by their partner. Most of the teen mothers had conflicts with their partners, and most of them refused the pregnancy. Only one out of 20 interviewed teen mothers came with her partner for the first visit, and the guy escaped the area.

*They required that I bring my husband and I explained to them that I was single and not married, they asked me to bring paper from my cell to prove that I don’t have a husband. After getting the required paper, they assessed me again and commended that I have to bring my husband, I communicated with him but he declined. When I went back and said that he refused, they reassessed and registered me in the program.* Site 3, P2

Some study participants reported being asked a community health insurance while they could not afford it. For instance, one participant reported, *“You know, it’s very known that you can’t be received without a health insurance and I didn’t have it. So, I had to go back to the community health worker”* Site 1, P2.

Fear of being stigmatized by other adult mothers at the healthcare setting was also reported by some study participants*, “I was not comfortable going to the clinic because it happened once other mothers asked me many questions. For example, one mother asked me how I got pregnant at a young age. I felt embarrassed and frustrated”* Site 3, P3. These participants added that they were not comfortable going to antenatal care since they would meet with their neighbors and family’s friends, whom they didn’t want to meet while they were pregnant. A few other participants reported the healthcare providers’ negative attitudes towards teen mothers, such as being rude, scolding, and blaming them.

For example, a study participant explained, "A nurse was giving me instructions and I could not follow them well, and she rudely said, It’s not surprising that you are pregnant because you don’t listen. Site 1, **P**1 Another participant added, "A nurse looked at me for a few seconds and asked me: How could you do this at this age? This is inappropriate at your age". Site 2, P2.

This illustrates that pregnant adolescents who accessed health services faced many challenges related to health centers' guidelines and procedures. These challenges included increased community stigma even in the healthcare environment and healthcare providers’ negative attitudes towards teen mothers. These challenges often cause pregnant adolescents to avoid seeking health services since they expressed not feeling comfortable, leading to poor health outcomes for both mother and child.

**Discussion**

This study aimed to explore and describe the consequences of adolescent childbearing in Rwanda. From the participants’ narratives, we identified health outcomes, socio-economic consequences, and access to healthcare services.

Our findings revealed that teen mothers faced health problems, including physical and psychological ones, as a result of teenage pregnancy. Physical outcomes include signs and symptoms of anemia, high blood pressure, and prolonged labor. Adolescent mothers (aged 10–19 years) are more likely to develop eclampsia, puerperal endometritis, and systemic infections, and their babies are more likely to have low birth weights, preterm births, and severe birth defects (WHO, 2023). These outcomes highlight the importance of comprehensive reproductive health education and healthcare access for teenagers.

A few participants experienced mental health problems, including difficulty accepting they were pregnant, fear of disclosure, intense stress, anxiety and depression. These findings are consistent with a systematic review that has been conducted in sub-Saharan African countries to assess the mental health problems and gaps in service provision experienced by pregnant teens and young women (Mutahi et al., 2022). In this review, depression was the most studied mental health problem followed by substance use, stress and loss of self-esteem. In Rwanda, a recent study found that 48% of teen mothers develop post-partum depression (Niyonsenga & Mutabaruka, 2020). In the Rwandan context, teen mothers develop mental health problems because they are less able to talk to people about their challenges (Coast et al., 2021). However, data in developed countries show that the rate of mental health problems is reportedly low among teen mothers (Wong et al., 2020), reflecting the available resources to support teen mothers. This implies that interventions are needed to prevent the high rates of depression among teenage mothers in LMICs.

In our study, a few teen mothers reported suicidal thoughts and others tried to commit suicide as a result of different challenges they faced after getting pregnant. Suicidal attempts are common in African settings (Mutahi et al., 2022). Different factors contribute to suicide among teen mothers, including lack of social support and perceived health status as negative (Li et al., 2021; Musyimi et al., 2020). Teen moms and young women in sub-Saharan Africa might also be more likely to engage in self-harm and suicidal behaviour due to the sociocultural values placed on them (Mutahi et al., 2022). These risk factors values should be taken into account when providing services to teen mothers.

Teen mothers in this study, because of the challenges they faced, they felt abortion as a solution. This finding is consistent with findings from another study in the Rwandan context which found abortion as a potential outcome among teen mothers due to paternity denial (Coast et al., 2021). **Data from 2019 indicate that over half of unintended pregnancies among adolescent girls aged 15-19 result in abortions, usually performed in unsafe conditions in LMICs (Sully et al., 2019). Our study did not focus on who attempted to abort or had aborted. Therefore, future studies should explore abortion as one of the potential pregnancy resolutions among teen mothers and how interventions can be developed to prevent unsafe abortions.**

In the current study, some teen mothers reported several socio-economic consequences as a result of teenage pregnancy. They mentioned that they do not receive support from families and partners which exacerbate their financial situations. These findings are consistent with another study which has been conducted in Kenya (Kumar et al., 2018). This study found that parents of the teen mother or of the biological father provide negligible support. Despite their sympathy for their daughters' pregnancies, the mothers could not support them due to food shortages and lack of resources. Developing and implementing strategies to support adolescent mothers, especially those with few social supports, is an urgent need.

Perceived stigma and discrimination were also reported by most teen mothers in this study. They reported shrinking of the social world and fear of being seen outside their home places. Others reported a break in relationships with their close friends while others had conflicts with their parents especially fathers. Social stigma attached to teenage pregnancy was reported in other African settings including Rwanda (Coast et al., 2021; Kumar et al., 2018). Culturally, in most countries, it is highly stigmatized to engage in premarital sexual activity, and pregnancy disclosure implies concurrent sexual activity, whether consented to or not (Coast et al., 2021; Erasmus et al., 2020).

Teen mothers voiced they encountered problems in their family relationships such as quarrels, misunderstandings between parents, family separation, family conflicts and were found to be the first source of psychological problems of teen mothers after getting pregnancy. The announcement of a pregnancy by a teen can cause arguments, quarrels, and strained relations between the teen and her parents. These young women may even leave the house due to fear of physical aggression due to verbal assaults and the imposition of abortions (Araújo Maranhão et al., 2018).

While all governments are required by international law to provide an education for all children, some African countries currently prohibit pregnant teens and mothers from attending school (Tull, 2020). In Rwanda, pregnant teens and teen mothers are allowed to attend schools, however, teen mothers in this study reported that they experience some school interruptions and drop-outs. The prevalence of drop out from school among teen mothers continues to be worrisome in African countries (Sobngwi-Tambekou et al., 2022; Stoner et al., 2019). It is necessary to tailor programs specific to the needs of pregnant teen who drop out of school, those in school but at risk of becoming pregnant, and those in school but become pregnant.

Teen mothers in this study reported struggling to care for themselves and their children and to cope with economic insecurity because they couldn't receive any or limited support from their families. These findings were also reported in other countries and Rwanda (Coast et al., 2021; Ntshayintshayi et al., 2022). Moreover, impregnating a teen in Rwanda is illegal whether a child consents or not (Rwanda Official Gazette No. Special of 27/09/2018, Section 6, 2018). Therefore, the perpetrators choose to abandon the teen mothers and sometimes flee the country (UNFPA, 2021), which add a financial burden to the teen mother since she cannot receive support from the partner. Policies and laws should be re-worked on to ensure they support the interests of the teen mothers and their babies.

Some study participants reported some barriers to accessing healthcare services, including being requested to bring their partners to the health facility in ANC at the first visit. These findings were also reported in previous studies (Apolot et al., 2020; Hackett et al., 2019; Påfs et al., 2016). Another issue reported was the need to present community health insurance while they could not afford it. Some teen mothers highlighted that they were from low-income families who could not afford to pay 3000 Rwandan francs, which is the cost of a health insurance policy per person. Moreover, to be given health insurance, all family members should pay (Rwanda Social Security Board (RSSB), 2023), which is an added barrier for teen mothers who are ostracized in their families. The community health insurance policy should consider the particularities of teen mothers to ensure they access services.

In this study, teen mothers shun health services due to fear of being stigmatized by other adult mothers in the healthcare setting. Teen mothers were accused of violating the age norms of parenting. These findings implicate that teen mothers should not share the waiting room and antenatal care with adult mothers to ensure privacy. A few other participants reported the healthcare providers’ negative attitudes towards teen mothers, such as being rude, scolding, and being blamed for getting pregnant at an early age. These findings corroborate other studies that were conducted in different settings (Apolot et al., 2020; Kola et al., 2020; Mweteni et al., 2021).

This study has some limitations. First, it was conducted in one district, so the findings may not be generalized to all teen mothers in the whole country. Second, the sample size included teen mothers from low-income families without social support. Participants with social support or from higher-income families might have different experiences. Third, almost all study participants lived in rural areas. This may have impacted the study results as rural areas have different resources and support systems compared to urban areas. Finally, the study only included teen mothers with primary education, so the findings may not be applicable to those with secondary education. Future research should employ a mixed methods approach to better capture the consequences of teen mothers with diverse educational attainments. Additionally, research should investigate the impact of social support and income on teenage pregnancy consequences in different communities. Finally, research should include more diverse participants to understand the topic better.

**Conclusion**

This study revealed that being a teenage mother in the Rwandan context is associated with several challenges, including poor health outcomes, socio-economic consequences, and structural barriers to accessing healthcare services. To prevent teenage pregnancies, different stakeholders should also create awareness about the consequences of teenage pregnancies and empower adolescents with the necessary knowledge and skills before reaching the adolescent stage. To prevent poor health outcomes such as physical health and mental health problems, teenage mothers need adequate access to healthcare services. This includes providing teenage mothers with adequate health information and access to community resources and support services. To cope with the socio-economic challenges of becoming a young parent, teenage mothers should be provided access to economic and social services, especially those from rural and low-income families. Lastly, policies and laws should be revised to address structural barriers to healthcare services. This includes avoiding requesting them to present their partners, providing teenage mothers with free health insurance, establishing private antenatal areas, and providing training to healthcare providers on how to dismantle their negative attitudes towards adolescent mothers.

**Acknowledgements**

We acknowledge the 20 teen mothers for participating in this study. Pre-Publication Support Service (PREPSS) supported the development of this manuscript by providing author training, as well as pre-publication peer-review and copy editing.

**Authors’ contributions**

IT, JR, AN, AN, VB, and BN designed the study. IT, JR, AN, and VB collected the data. IT, JR, and AN analyzed the data and wrote the first draft of the manuscript. All authors read and approved the final manuscript.

**Funding**

This work was supported by the Center for International Reproductive Health Training at the University of Michigan, US.

**Availability of data and materials**

The datasets analyzed during the current study are available from the corresponding author upon reasonable request.

**Declarations for ethics approval and consent to participate**

The Institute Review Board from College of Medicine and Health Sciences, University of Rwanda (No 302/CMHS IRB/2022) and Gatsibo District (Ref No: 1158/07/05/03/01 M) Approved the study and provided the written ethical clearance. Prior to data collection all participants signed the assent and their legal guardian signed the consent to participant in the study. The research followed the determinations of the Declaration of Helsinki, as well as the norms that regulate the research involving human beings in Rwanda.

**Consent for publication**

Not applicable.

**Competing interests**

The authors declare that they have no conflicts of interest.

**References**

Ajala, A. O. (2014). Factors associated with teenage pregnancy and fertility in Nigeria. *Issn*, *5*(2), 2222–1700.

Ajayi, A. I., Odunga, S. A., Oduor, C., & Ouedraogo, R. (2021). *" I was tricked ": understanding reasons for unintended pregnancy among sexually active adolescent girls*. 1–11. https://doi.org/10.1186/s12978-021-01078-y

Apolot, R. R., Tetui, M., Nyachwo, E. B., Waldman, L., Morgan, R., Aanyu, C., Mutebi, A., Kiwanuka, S. N., & Ekirapa, E. (2020). Maternal health challenges experienced by adolescents; could community score cards address them? A case study of Kibuku District– Uganda. *International Journal for Equity in Health*, *19*(1), 1–12. https://doi.org/10.1186/s12939-020-01267-4

Araújo Maranhão, T., dos Santos Sales, S., Lúcia Duarte Pereira, M., Ibiapina Cordeiro, L., Suellen Pires de Sousa, C., & Mld, P. (2018). Family and Social Attitudes and Reactions Before Pregnancy in Adolescence. *Journal of Nursing UFPE*, *12*(4), 840–848. https://doi.org/https://doi.org/10.5205/1981-8963-v12i3a234547p840-848-2018

Bahamondes, L. (2018). Long term social consequences of adolescent pregnancy. *An International Journal Obstetrics and Gynaecology*, 360–367. https://doi.org/doi.org/10.1111/1471-0528.15428

Braun, V., & Clarke, V. (2006). Using thematic analysis in psychology. *Qualitative Research in Psychology*, *3*(2), 77–101. https://doi.org/https://doi.org/10.1191/1478088706qp063oa

Chung, H. W., Kim, E. M., & Lee, J.-E. (2018). Comprehensive understanding of risk and protective factors related to adolescent pregnancy in low- and middle-income countries : A systematic review. *Journal of Adolescence*, *69*, 180–188. https://doi.org/10.1016/j.adolescence.2018.10.007

Coast, E., Merci, M., Roberte, M., Ngabonzima, E., Pereznieto, P., Buzby, S., Dutton, R., & Baird, S. (2021). ‘ If She ’ s Pregnant , then that Means that Her Dreams Fade Away ’: Exploring Experiences of Adolescent Pregnancy and Motherhood in Rwanda. *The European Journal of Development Research*, *33*(5), 1274–1302. https://doi.org/10.1057/s41287-021-00438-5

Creswell, J. . (2014). *Research Design: Qualitative, Quantitative and Mixed Methods Approaches*. SAGE Publications.

Darroch, J. E., Woog, V., Bankole, A., & Ashford, L. S. (2016). Adding It Up: Costs and Benefits of Meeting the Contraceptive Needs of Adolescents. In *Guttmacher Institute*. www.guttmacher.org/report/%0Aadding-it-meeting-contraceptive-needs-of-adolescents

Erasmus, M. O., Knight, L., & Dutton, J. (2020). Barriers to accessing maternal health care amongst pregnant adolescents in South Africa : a qualitative study. *International Journal of Public Health*, *65*(4), 469–476. https://doi.org/10.1007/s00038-020-01374-7

Gurung, R., Målqvist, M., Hong, Z., Poudel, P. G., Sunny, A. K., Sharma, S., Mishra, S., Nurova, N., & Kc, A. (2020). The burden of adolescent motherhood and health consequences in Nepal. *BMC Pregnancy and Childbirth*, *20*(1), 1–7. https://doi.org/10.1186/s12884-020-03013-8

Guttmacher Institute. (2015). *Adolescent Pregnancy and Its Outcomes Across Countries* (Issue August).

Hackett, K., Lenters, L., Vandermorris, A., Lafleur, C., Newton, S., Ndeki, S., & Zlotkin, S. (2019). How can engagement of adolescents in antenatal care be enhanced ? Learning from the perspectives of young mothers in Ghana and Tanzania. *BMC Pregnancy and Childbirth*, *19*(184), 1–12. https://doi.org/https://doi.org/10.1186/s12884-019-2326-3 (2019)

Karaçam, Z., Kizilca, D., & Demir, R. (2021). The impact of adolescent pregnancy on maternal and infant health in Turkey : Systematic review and meta-analysis. *Journal of Gynecology Obstetrics and Human Reproduction*, *50*(102093), 1–15. https://doi.org/10.1016/j.jogoh.2021.102093

Kirbas, A., Gulerman, H. C., & Daglar, K. (2016). Pregnancy in Adolescence: Is it an obstetrical risk? *Journal of Pediatric and Adolescent Gynecology*. https://doi.org/10.1016/j.jpag.2015.12.010

Kola, L., Bennett, I. M., Bhat, A., Ayinde, O. O., Oladeji, B. D., Abiona, D., Abdumalik, J., Faregh, N., Collins, P. Y., & Gureje, O. (2020). Stigma and utilization of treatment for adolescent perinatal depression in Ibadan Nigeria. *BMC Pregnancy and Childbirth*, *20*(294), 1–8. https://doi.org/https://doi.org/10.1186/s12884-020-02970-4 (2020)

Kumar, M., Huang, K. Y., Othieno, C., Wamalwa, D., Madeghe, B., Osok, J., Kahonge, S. N., Nato, J., & McKay, M. M. K. (2018). Adolescent Pregnancy and Challenges in Kenyan Context: Perspectives from Multiple Community Stakeholders. *Global Social Welfare*, *5*(1), 11–27. https://doi.org/10.1007/s40609-017-0102-8

Lebni, J. Y., Farahani, F. K., Solhi, M., & Azar, F. E. F. (2021). Causes and grounds of childbirth fear and coping strategies used by kurdish adolescent pregnant women in iran: A qualitative study. *Journal of Reproduction and Infertility*, *22*(1), 47–56. https://doi.org/10.18502/jri.v22i1.4995

Li, J., Imam, S. Z., Jing, Z., Wang, Y., & Zhou, C. (2021). Suicide attempt and its associated factors amongst women who were pregnant as adolescents in Bangladesh: a cross-sectional study. *Reproductive Health*, *18*(1), 1–9. https://doi.org/10.1186/s12978-021-01127-6

Musyimi, C. W., Mutiso, V. N., Nyamai, D. N., Ebuenyi, I., & Ndetei, D. M. (2020). Suicidal behavior risks during adolescent pregnancy in a low-resource setting: A qualitative study. *PLoS ONE*, *15*(7), 1–11. https://doi.org/10.1371/journal.pone.0236269

Mutahi, J., Larsen, A., Cuijpers, P., Peterson, S. S., Unutzer, J., McKay, M., John-Stewart, G., Jewell, T., Kinuthia, J., Gohar, F., Lai, J., Wamalwa, D., Gachuno, O., & Kumar, M. (2022). Mental health problems and service gaps experienced by pregnant adolescents and young women in Sub-Saharan Africa: A systematic review. *EClinicalMedicine*, *44*(101289), 101289. https://doi.org/10.1016/j.eclinm.2022.101289

Mweteni, W., Kabirigi, J., Id, D. M., Laisser, R., Yohani, V., Shabani, G., Shayo, P., Brenner, J., & Id, K. C. (2021). Implications of power imbalance in antenatal care seeking among pregnant adolescents in rural Tanzania : A qualitative study. *PLoS ONE*, *16*(6), 1–19. https://doi.org/10.1371/journal.pone.0250646

Ngoda, O. A., Mboya, I. B., Mahande, M. J., Msuya, S. E., & Renju, J. (2021). Trends and factors associated with repeated adolescent pregnancies in tanzania from 2004-2016: Evidence from tanzania demographic and health surveys. *Pan African Medical Journal*, *40*. https://doi.org/10.11604/PAMJ.2021.40.162.29021

Niyonsenga, J., & Mutabaruka, J. (2020). Factors of postpartum depression among teen mothers in Rwanda : a cross- sectional study. *Journal of Psychosomatic Obstetrics & Gynecology*, 1–5. https://doi.org/10.1080/0167482X.2020.1735340

Nkurunziza, A., Endert, N. Van, Bagirisano, J., Hitayezu, J. B., Dewaele, S., Tengera, O., & Jans, G. (2020). Breaking barriers in the prevention of adolescent pregnancies for in-school children in Kirehe district ( Rwanda ): a mixed-method study for the development of a peer education program on sexual and reproductive health. *Reproductive Health*, *17*(137), 1–8.

Ntshayintshayi, P. N., Sehularo, L. A., Mokgaola, I. O., & Sepeng, N. V. (2022). Exploring the psychosocial challenges faced by pregnant teenagers in Ditsobotla subdistrict. *Health SA Gesondheid*, *27*, 1–8. https://doi.org/10.4102/hsag.v27i0.1880

Påfs, J., Musafili, A., Binder-finnema, P., Klingberg-allvin, M., Rulisa, S., & Essén, B. (2016). Beyond the numbers of maternal near-miss in Rwanda – a qualitative study on women ’ s perspectives on access and experiences of care in early and late stage of pregnancy. *BMC Pregnancy and Childbirth*, 1–11. https://doi.org/10.1186/s12884-016-1051-4

Rwanda Official Gazette no. Special of 27/09/2018, Section 6, 17 (2018).

Rwanda Social Security Board (RSSB). (2023). *Community Based Health Insurance (CBHI) Scheme*. RSSB. https://www.rssb.rw/scheme/cbhi-scheme

Sobngwi-Tambekou, J. L., Tsague-Agnoux, M., Fezeu, L. K., & Ndonko, F. (2022). Teenage childbearing and school dropout in a sample of 18,791 single mothers in Cameroon. *Reproductive Health*, *19*(1), 1–9. https://doi.org/10.1186/s12978-021-01323-4

Stoner, M. C. D., Rucinski, K. B., Edwards, J. K., Selin, A., Hughes, J. P., Wang, J., Agyei, Y., Gomez-Olive, F. X., MacPhail, C., Kahn, K., & Pettifor, A. (2019). The Relationship Between School Dropout and Pregnancy Among Adolescent Girls and Young Women in South Africa: A HPTN 068 Analysis. *Health Education and Behavior*, *46*(4), 559–568. https://doi.org/10.1177/1090198119831755

Sully, E. A., Biddlecom, A., Darroch, J., Riley, T. S., Ashford, L. S., Lince-Deroche, N., Firestein, L., & Murro, R. (2019). *Adding it up: Investing in Sexual and Reproductive Health*. http://www.unfpa.org/webdav/site/global/shared/documents/publications/2009/adding_it_up_report.pdf

Svanemyr, J. (2020). Adolescent pregnancy and social norms in Zambia. *Culture, Health and Sexuality*, *22*(6), 615–629. https://doi.org/10.1080/13691058.2019.1621379

Tull, K. (2020). *Consequences for adolescents when they become pregnant , and become mothers. K4D Helpdesk Report 882.*

UNFPA. (2021). *Supporting young mothers in Nyamasheke District to build new lives*. UNFPA. https://rwanda.unfpa.org/en/news/supporting-young-mothers-nyamasheke-district-build-new-lives

UNFPA. (2022). *Nearly half of all pregnancies are unintended—a global crisis, says new UNFPA report*.

Uwizeye, D., Muhayiteto, R., Kantarama, E., Wiehler, S., & Murangwa, Y. (2020). Prevalence of teenage pregnancy and the associated contextual correlates in Rwanda. *Heliyon*, *6*(10), e05037. https://doi.org/10.1016/j.heliyon.2020.e05037

Vieira Martins, M., Karara, N., Dembiński, L., Jacot-Guillarmod, M., Mazur, A., Hadjipanayis, A., & Michaud, P.-A. (2023). Adolescent pregnancy: An important issue for paediatricians and primary care providers—A position paper from the European academy of paediatrics. *Frontiers in Pediatrics*, *11*, 1119500.

WHO. (2019). *Maternal mortality: evidence brief*. World Health Organization.

WHO. (2023). *Adolescent pregnancy*. WHO. https://www.who.int/news-room/fact-sheets/detail/adolescent-pregnancy#:~:text=Adolescent mothers (aged 10–19,birth and severe neonatal condition.

Wong, S. P. W., Twynstra, J., Gilliland, J. A., Cook, J. L., & Seabrook, J. A. (2020). Risk Factors and Birth Outcomes Associated with Teenage Pregnancy: A Canadian Sample. *Journal of Pediatric and Adolescent Gynecology*, *33*(2), 153–159. https://doi.org/10.1016/j.jpag.2019.10.006

Worku, M. G., Tessema, Z. T., Teshale, A. B., Tesema, G. A., & Yeshaw, Y. (2021). Prevalence and associated factors of adolescent pregnancy ( 15 – 19 years ) in East Africa : a multilevel analysis. *BMC Pregnancy and Childbirth*, *21*(53), 1–8.
