# Supplementary material for: “My friends said they should no longer be with a b*tch like me…”: A qualitative study to explore the consequences of adolescent childbearing among teen mothers in Gatsibo district, Rwanda
Source: PLOS Glob Public Health. 2024 Sep 11;4(9):e0003716. doi: 10.1371/journal.pgph.0003716 (PMC11389937; doi:10.1371/journal.pgph.0003716)
Supplement: Attachment — Submitted filename: Response to Reviewers.docx [file pgph.0003716.s002.docx]

**Responses to reviewers**

Reviewer#1

On behalf of the authors, I would like to express my deepest gratitude for your valuable manuscript comments. Your thorough and insightful feedback has been instrumental in helping us improve the quality of our work. We greatly value your expertise and guidance in reviewing our manuscript. Your careful reading and attention to detail have provided us with valuable insights into various aspects of our paper, including clarity, organization, and presentation.

| **#** | **Comments** | **Responses** |
| --- | --- | --- |
|  | **GENERAL** | |
|  | General comment reviewer #1: Whilst this paper addresses an important and relevant topic, there are numerous errors and typos, which detract from the content. I believe that the topic and the data are important and should be published, however in order to get it to a publishable standard, the paper would need a thorough copy edit. In the specific comments below I picked up some mistakes and typos, but I am sure there are some I missed. An English language editor should do a detailed edit to ensure these are captured. | Thank you very much for your feedback. This paper was thoroughly edited by the Pre-Publication Support Service (PREPSS) at the University of Michigan/USA, which provides scientific communication training to health researchers and instructors from around the globe.  For more information, this is their website:  <https://sites.google.com/umich.edu/prepss> |
|  | **ABSTRACT** | |
|  | Line 37: “descriptive” doesn’t need to be capitalized | The term was revised to ‘descriptive’ |
|  | Line 38: “for interviewing”? –revise sentence | The sentence structure was revised to “for in- depth interview. |
|  | Line 39: “conversations were conducted in Kinyarwanda and audio recorded” | The sentence structure changed as advised |
|  | Line 39: translated into English transcripts? What was the translation process – provide more detail | Detail were provided as follow: “The research team, fluent in both English and Kinyarwanda, carefully listened to each recording and verbatim translated them into English ensuring they are clear and understandable”. |
|  | Line 52: did the study examine consequences? | Yes , our study explored the consequences of adolescent childbearing among teen mothers in Gatsibo district, Rwanda |
|  | **BACKGROUND** | |
|  | Line 85-87: “Despite these alarming statistics, little is known about the consequences associated with teenage pregnancies among these female adolescents to understand the full scope of the issue better.” – something missing from this sentence? Needs to be revised | The sentence structure was changed to “Despite these alarming statistics, little is known about the consequences associated with teenage pregnancies among these female adolescents. Further research is needed to better understand the full scope of the issue and inform policy decisions and interventions to support these young women.” |
|  | Line 95: capitalise “Eastern” if pronoun | The term was revised |
|  | Line 109: who is “us”? – preferable to write in third person – i.e. “the research team” – same comment with “we” in rest of paragraph | The paragraph revised. |
|  | Line 144: all interviews were conducted in a 2 day period?? | No it was a typing error. Data collection started August 30, 2022, and ended September 30, 2022. Error addressed. |
| **FINDINGS** | | |
|  | Line 160: where does the data in the table come from? Was a demographic questionnaire also administered? If so, describe it in methods | We asked the socio-demographic data before each interview. |
|  | Line 171: skin turning yellow meaning anaemia or jaundice? | In black skinned colored people, it is yellow. |
|  | Line 218: what is meant by “loss of future”? | In the study's context, a teen mother may experience a loss of the future when she faces unexpected circumstances that disrupt her plans or goals. |
|  | Line 222: what is meant by “disappointed in their families” ? | Sentence structure changed |
|  | Lines 234-240: sentences need revision – do not make sense and require punctuation | The sentence was changed to make it clear to the readers |
|  | For example Line 234: “their pregnancy is essence”? | The word essence was removed. We changed the sentence structure. |
|  | Line 237: “most the teen fathers are the first to traumatize their daughters but fewest mothers also have been found” ?? – does not make sense | The sentence structure was modified. |
|  | Line 251: refrain from using the term ‘prostitutes’ unless it is a direct quotation indicated by quotation marks. The term sex worker is more appropriate | We changed the word to sex workers |
|  | Lines 251-252: “misbehaved girls, they talk on their back saying none can give good advices as they got pregnant early” – does not make sense | We changed the sentence structure to make it clear |
|  | Line 271: “the hiring people” – do you mean employers? | We changed the word to employers |
|  | Line 287: “and the guy escaped the area” ?? | We changed the sentence structure. |
|  | Line 293: “Some study participants reported being asked a community health insurance while they could not afford it”- word missing? ? | We added to pay for it. |
|  | Line 343: “they faced, they felt” – cut the 2nd they - revise | The sentence was revised |
|  | Line 366: “voiced that they encountered” – revise | The sentence was revised |

Reviewer#2

Thank you very much for your time and efforts to provide us the constructive feedback. We have taken your feedback into consideration and have revised the manuscript accordingly. We have addressed each of your comments and suggestions, striving to make the necessary changes to improve the overall quality of our work. We are confident that the updated version reflects your valuable input. Once again, thank you for your time and effort in reviewing our manuscript. Your assistance has been invaluable, and we appreciate the rigor and fairness with which you carried out the review.

| **#** | **Comments** | **Responses** |
| --- | --- | --- |
|  |  |  |
|  | 1.There are several grammatical and punctuation mistakes. I would recommend authors to have their manuscript proof-read by a native speaker or use online services to rectify these. | Thank you very much for your feedback. The Pre-Publication Support Service (PREPSS) at the University of Michigan/USA, which provides scientific communication training to health researchers and instructors from around the globe has thoroughly edited this paper and corrected all the observed grammatical and punctuation mistake.  For more information, this is their website:  <https://sites.google.com/umich.edu/prepss> |
|  | 2. Some parts of the abstract and introduction need better context setting in terms of regions the authors want to focus on. Please see the comments in the attached file. | The comments in the file was well addressed and we focused more on the LMICs and more specifically Rwanda context |
|  | 3. The method section needs some clarifications and more details on the data collection for example the positionality of the authors in comparison to the participants. Please see the comments in the attached file. | Comments on the method section were addressed as advised. In relation to the positionality of authors, we described that participants met with two experienced female co-investigators in a private room for an audio-recorded interview. We also mentioned in the method section that Daily memoing practice was done to ensure that co-investigator personal perspectives would not influence the interpretation of data |
|  | 4.In the results section, please follow the same structure with an explanation of the main themes then evidence in form of quotes. Also add the number of participants who reported a particular consequence. Some paragraphs have these counts and others do not. | We revised the explanations. We followed the same structure and added the number of the participants. |
|  | 5.The discussion is well written but need some more reflection on topics like role of men, partners, boys in the broader scope of intervention design and supporting girls. The topic of marital status should also be discussed within the SSA context and in other LMICs. For example, adolescent pregnancy happens within the context of marriage in south asia while this is not the case in this study. | The role of men and boys is reflected on in the discussion section. |
|  | 6. a section recommendations on how to support girls is missing. Also comment on the widespread application of gender transformative interventions or comprehensive sexual health education and how they can potentially support girls in this context. | We added in the recommendation section that “Governments and organizations should develop and implement gender-sensitive policies and programs to support adolescent mothers” and provided additional details throughout the discussion section. |
